# CsWRKY11 cooperates with CsNPR1 to regulate SA-triggered leaf de-greening and reactive oxygen species burst in cucumber

**DOI:** 10.1186/s43897-024-00092-5

**Published:** 2024-05-22

**Authors:** Dingyu Zhang, Ziwei Zhu, Bing Yang, Xiaofeng Li, Hongmei Zhang, Hongfang Zhu

**Affiliations:** 1 Shanghai Key Laboratory of Protected Horticultural Technology, Horticultural Research Institute, Shanghai Academy of Agricultural Sciences, 1000 Jinqi Road, Shanghai, 201403 China; 2 State Key Laboratory of Genetic Engineering and Fudan Center for Genetic Diversity and Designing Agriculture, School of Life Sciences, Fudan University, Shanghai, 200438 China

**Keywords:** Salicylic acid, CsNPR1, CsWRKY11, Chlorophyll degradation, ROS burst

## Abstract

Salicylic acid (SA) is a multi-functional phytohormone, regulating diverse processes of plant growth and development, especially triggering plant immune responses and initiating leaf senescence. However, the early SA signaling events remain elusive in most plant species apart from Arabidopsis, and even less is known about the multi-facet mechanism underlying SA-regulated processes. Here, we report the identification of a novel regulatory module in cucumber, CsNPR1-CsWRKY11, which mediates the regulation of SA-promoted leaf senescence and ROS burst. Our analyses demonstrate that under SA treatment, CsNPR1 recruits CsWRKY11 to bind to the promoter of *CsWRKY11* to activate its expression, thus amplifying the primary SA signal. Then, CsWRKY11 cooperates with CsNPR1 to directly regulate the expression of both chlorophyll degradation and ROS biosynthesis related genes, thereby inducing leaf de-greening and ROS burst. Our study provides a solid line of evidence that CsNPR1 and CsWRKY11 constitute a key module in SA signaling pathway in cucumber, and gains an insight into the interconnected regulation of SA-triggered processes.

**Supplementary Information:**

The online version contains supplementary material available at 10.1186/s43897-024-00092-5.

## Core

CsNPR1 recruits CsWRKY11 to bind to the promoter of *CsWRKY11*, thus promoting the self-activation of CsWRKY11; CsWRKY11 further effects synergistically with CsNPR1 to regulate SA-triggered leaf de-greening and ROS burst in cucumber.

## Gene & accession numbers

CsWRKY11 (CsaV3_2G017760), CsWRKY12 (CsaV3_2G032460), CsWRKY27 (CsaV3_3G033000), CsWRKY28 (CsaV3_3G033350), CsWRKY35 (CsaV3_4G006480), CsWRKY41 (CsaV3_5G011080), CsWRKY49 (CsaV3_6G042200), CsWRKY50 (CsaV3_6G042280), CsWRKY55-like (CsaV3_5G011070), CsWRKY2 (CsaV3_1G004720), CsWRKY5 (CsaV3_1G032000), CsWRKY10 (CsaV3_2G017720), CsWRKY19 (CsaV3_3G008170), CsWRKY30 (CsaV3_3G047140), CsWRKY37 (CsaV3_4G034570), CsWRKY40 (CsaV3_5G011060), CsWRKY52 (CsaV3_6G048830), CsWRKY59 (CsaV3_7G025370), CsNPR1 (CsaV3_4G007550). Raw sequencing data have been deposited to NCBI Sequence Read Archive (SRA), accession numbers PRJNA771246.

## Introduction

Leaf senescence is an integral part of plant development, during which plant cells undergo a degenerative process involves a rapid but coordinated breakdown of chlorophylls, proteins, and other macromolecules (Kuai et al. 2018). Released nutrients from senescent leaves are mainly transported to the newborn or reproductive organs, which makes a substantial contribution to grain yield formation as well as environmental adaptability of crop plants (Lim et al. 2007; Guiboileau et al. 2012; Guo et al. 2021). Chlorophyll degradation, leading to a visible de-greening symptom, is the prominent biochemical event of leaf senescence. The biochemical pathway of chlorophyll degradation has been extensively investigated, resulting in the identification of key chlorophyll catabolic enzymes (CCEs), including chlorophyll b reductase (NYC1/NOL), 7-hydroxymethyl chlorophyll a reductase (HCAR), Mg-dechelatase (NYE1/SGR1), pheophorbide hydrolase (PPH), pheophorbide an oxygenase (PAO), red chlorophyll catabolite reductase (RCCR) (Kuai et al. 2018; Guo et al. 2021). The senescing process also involves a burst of reactive oxygen species (ROS), i.e. superoxide anion (O_2_^·−^), hydrogen peroxide (H_2_O_2_), singlet oxygen (^1^O_2_), and hydroxyl radical (HO^·^). ROS have been identified as signal molecules to regulate plant responses to stresses, and recent studies also highlighted an importance of ROS in organelle-to-organelle or cell-to-cell signal communication (Mittler 2017; Mittler et al. 2022). Accumulation of intracellular ROS is a result of oxidation–reduction reaction in chloroplasts, mitochondria and peroxisomes, whereas apoplastic ROS, greatly contributing to plant defenses against pathogens, are mainly produced via the catalyzation of the plasma membrane-localized NADPH oxidases (respiratory burst oxidase homologues, RBOHs) (Zhang et al. 2020).

Both chlorophyll degradation and ROS burst are strictly monitored in plants, many senescence regulators, such as EIN3, ORE1, NAP, PIF4/5, ABF2/3/4, MYC2/3/4 and ANAC019/055/072, were shown directly regulating the expression of *chlorophyll catabolic genes* (*CCGs*) (Kuai et al. 2018). RBOHs-catalyzed ROS were reported being regulated by WRKY7/8/9/11, ERF74/75, and *ROOT HAIR DEFECTIVE SIX-LIKE 4* (*RSL4*) etc. (Adachi et al. 2015; Yao et al. 2017; Zhang et al. 2022b). Recently, we found CsEIN3 simultaneously regulates the expressions of both *CsCCGs* and *CsRBOHs*, leading to accelerated chlorophyll degradation and ROS metabolism in cucumber (Zhang et al. 2022a). Increasing evidence suggests that the defense-related phytohormone salicylic acid (SA) also plays a positive role in regulating leaf senescence (Morris et al. 2000; Breeze et al. 2011). Reducing endogenous SA content through overexpressing an SA hydrolase *NahG* significantly delays the process of leaf senescence; mutating the SA receptor NON-EXPRESSOR OF PATHOGENESIS-RELATED GENES1 (NPR1) to block SA signal transduction significantly inhibits the SA-induced leaf senescence, while mutating the *SA 3-HYDROXYLASE* (*S3H*) gene, which results in increased endogenous SA levels, significantly accelerates leaf senescence (Morris et al. 2000; Lim et al. 2007; Zhang et al. 2013); in addition, SA gradually accumulates in an age-dependent manner, implying that SA regulates natural senescence of plants (Morris et al. 2000; Breeze et al. 2011).

NPR1, the receptor of SA, is the hub component of SA signal, also acting as a transcription coactivator (Spoel et al. 2009; Ding et al. 2018). Nucleus-located NPR1 can recruit numerous transcription factors like TGAs, AtEIN3/AtEIL1, AtMYC2, AtbZIP28/60, AtTCP8/14/15, AtHSFA1, and PpMADS2 to bind to the promoters of SA responsive genes, thereby activating their expression in diverse plant species (Zhang et al. 1999; Després et al. 2000; Spoel et al. 2009; Wu et al. 2012; Jin et al. 2018; Lai et al. 2018; Olate et al. 2018; Huang et al. 2020; Nomoto et al. 2021; Kumar et al. 2022; Li et al. 2022). Nevertheless, in cucumber, the early event (s) of SA-triggered signaling, particularly the action mode of CsNPR1, still remain elusive. WRKY family is one of the largest plant-specific transcription factor families. It has proved that WRKYs participate in the response of plants to various stresses and phytohormone signal transduction (Jiang et al. 2017). WRKY3/4, WRKY18, WRKY22/29, WRKY33, WRKY38/62, WRKY46/53/70, and WRKY8/28/48 have been reported to regulate the SA-triggered defense process in *Arabidopsis thaliana* (Asai et al. 2002; Wang et al. 2006; Zheng et al. 2006; Eulgem and Somssich 2007; Kim et al. 2008; Xing et al. 2008; Pandey and Somssich 2009; Spoel et al. 2009; Hu et al. 2012; Jiang et al. 2017). In cucumber, WRKYs were reported participating in regulating its resistance to biotic and abiotic stresses (Chen et al. 2020; Yang et al. 2020; Meng et al. 2022). ABA mediates the cold response of cucumber via regulating the CsWRKY41/CsWRKY46-miR396b-5p-CsTPR module (Sun et al. 2022). CsWRKY50 positively regulates the immune response of cucumber plants to *Pseudoperonospora cubensis* through enhancing their antioxidation activity (Luan et al. 2019). However, thus far, the relevance of WRKY transcription factors in SA signaling has not been demonstrated in cucumber. Here, we reveal that the molecular mechanism of SA-induced leaf senescence involves a synergistic action of CsNPR1 and CsWRKY11 in cucumber. We demonstrate that SA-activated CsNPR1 translocated into nucleus and directly interacts with CsWRKY11 to activate the expression of *CsWRKY11*, thus leading to a primary activation of SA signaling; subsequently, CsNPR1 and CsWRKY11 synergistically promote chlorophyll degradation and ROS burst via up-regulating the expression of major *CsCCGs* and *CsRBOHB* in cucumber.

## Results

### SA promotes leaf senescence and ROS burst in cucumber

**Fig. 1:**
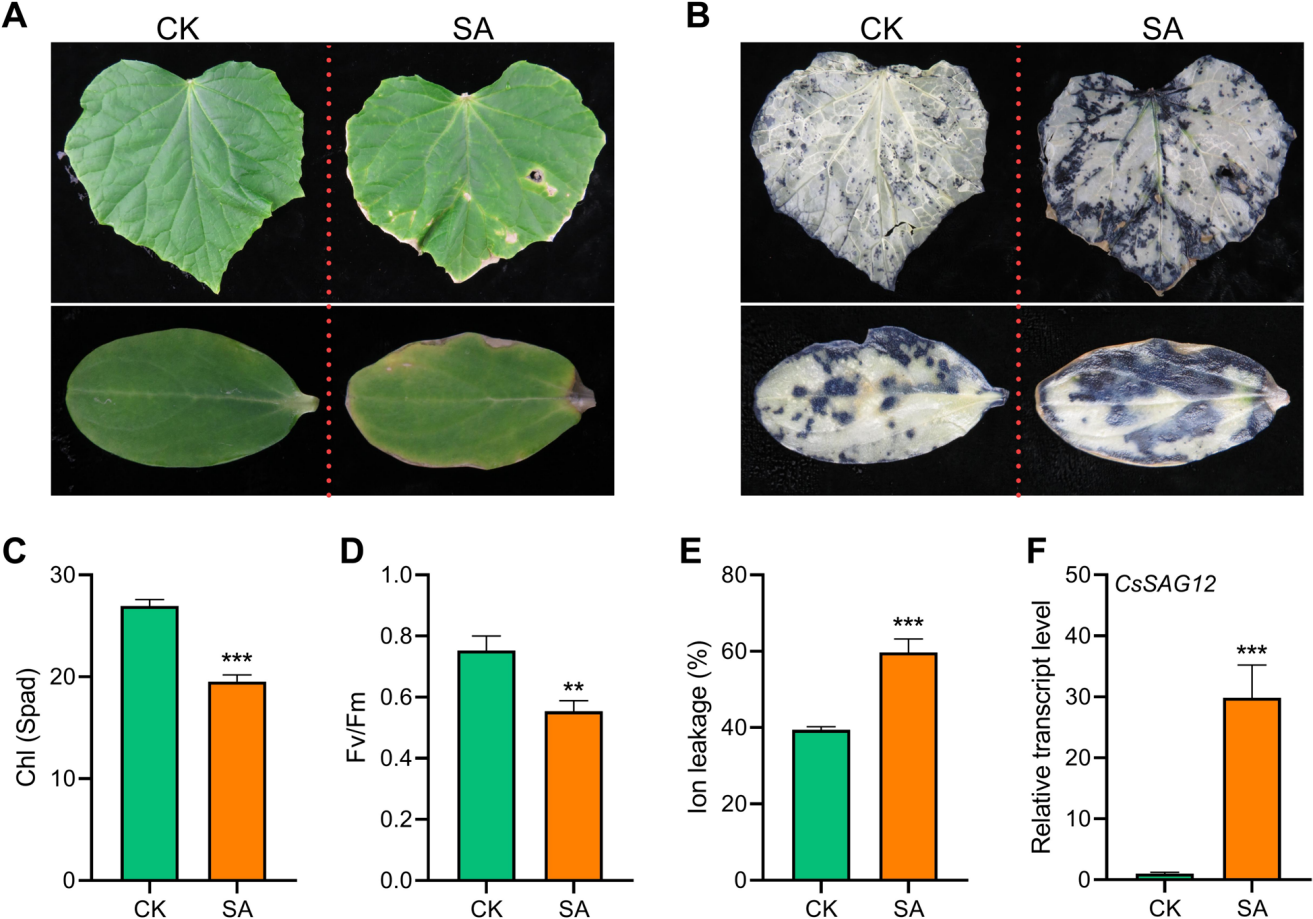
SA promotes leaf senescence and ROS burst in cucumber. **A** Phenotypes of SA and H_2_O (CK)-treated cucumber leaves and cotyledons. **B** Phenotypes of NBT-stained cucumber leaves and cotyledons after SA and CK treatments. **C**-**F** Chlorophyll content (**C**), Fv/Fm ratio (**D**), ion leakage (**E**), and relative transcript level of *CsSAG12* (**F**) in SA- and CK-treated cucumber leaves. In (**A**-**F**), detached leaves and cotyledons were floated on 1mM SA solution for 5 days, data are means ± SD (*n* = 3 biological replicates). *P < 0.05, **P < 0.01, ***P < 0.001 (*t*-test), primers are listed in Table S1

SA is not only a key defense-related phytohormone, but also play a crucial role in regulating plant growth and development (Peng et al. 2021). To evaluate the effects of SA treatment in cucumber, we treated cucumber leaves and cotyledons with SA solution. As shown in Fig. 1A, SA treatment significantly promoted the senescence process of cucumber leaves and cotyledons, initiating from their tips/margins. In addition, nitroblue tetrazolium (NBT) staining showed that the contents of O2^·−^ in the SA-treated leaves and cotyledons were obviously higher than those in control samples, consistent with the observed tissue necrosis phenotype (Fig. 1A, B). Analyses of their physiological and molecular phenotypes further demonstrated that SA treatment significantly incurred chlorophyll degradation, reduction of maximum photochemical efficiency of PSII (Fv/Fm), increasing of ion leakage, and upregulation of *CsSAG12* (Fig. 1C-F). These results suggest that SA treatment accelerates leaf senescence as well as ROS metabolism in cucumber.

### SA regulates chlorophyll degradation and ROS production by up-regulating *CsCCGs* and *CsRBOHB*

**Fig. 2:**
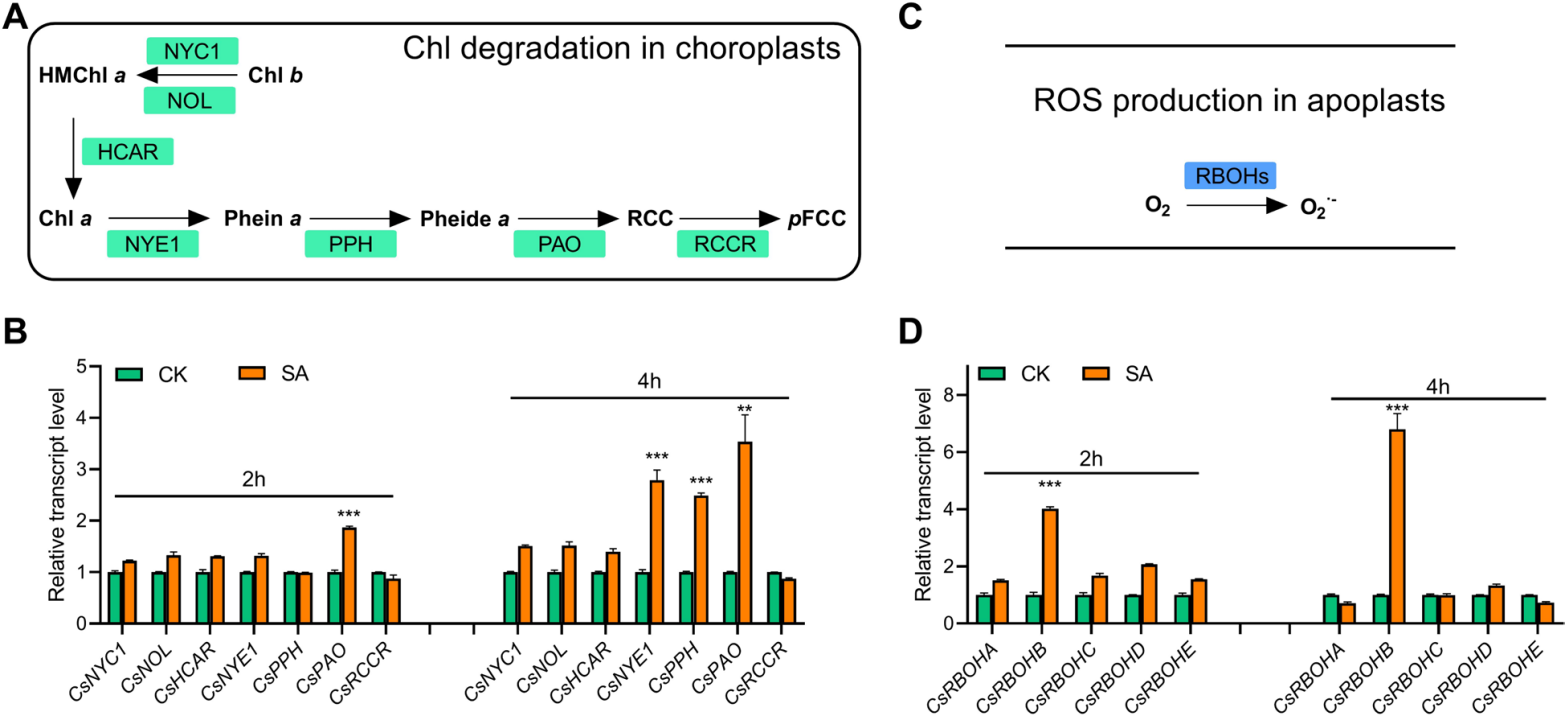
SA up-regulates the expression of *CsCCGs* and *CsRBOHB*. **A** The biochemical pathway of chlorophyll degradation in chloroplasts. **B** Time-course expression patterns of *CsCCGs* in response to SA treatment. **C** The biochemical pathway of ROS biosynthesis in apoplasts. **D** Time-course expression patterns of *CsRBOHs* in response to SA treatment. Data are means ± SD (*n* = 3 biological replicates), **P < 0.01, ***P < 0.001 (*t*-test), primers are listed in Table S1

Chlorophyll degradation is the prominent event of leaf senescence, which is sequentially catalyzed by a serious of chlorophyll catabolic enzymes (CCEs) (Kuai et al. 2018). ROS is generated, especially in apoplast, from a O_2_-to-O_2_^·−^ reaction under the catalyzation of RBOHs (Waszczak et al. 2018). Considering that SA treatment leads to significant chlorophyll degradation and ROS production, we further examined the involvement of specific *CsCCG*(*s*) and *CsRBOH*(*s*) in these regulatory processes. A time-course analysis of transcription showed that the expression of *CsPAO* was instantly induced, with a steady up-regulation afterward, while *CsNYE1* and *CsPPH* started to be significantly up-regulated in the late period of SA treatment, in comparison to a moderate induction of *CsNYC1*, *CsNOL*, and *CsHCAR*. Not surprisingly, *CsRCCR* showed no response at all to the treatment (Fig. 2A, B). Interestingly, among the five genes which encode ROS production enzymes, only *CsRBOHB* was robustly induced during the whole period of SA treatment (Fig. 2C, D). These results indicate that SA induction of chlorophyll degradation and ROS production largely depends on the up-regulation of *CsNYE1*/*CsPPH/CsPAO* and *CsRBOHB*, respectively in cucumber.

### SA signaling primarily activates* WRKY* transcription factors in cucumber

**Fig. 3:**
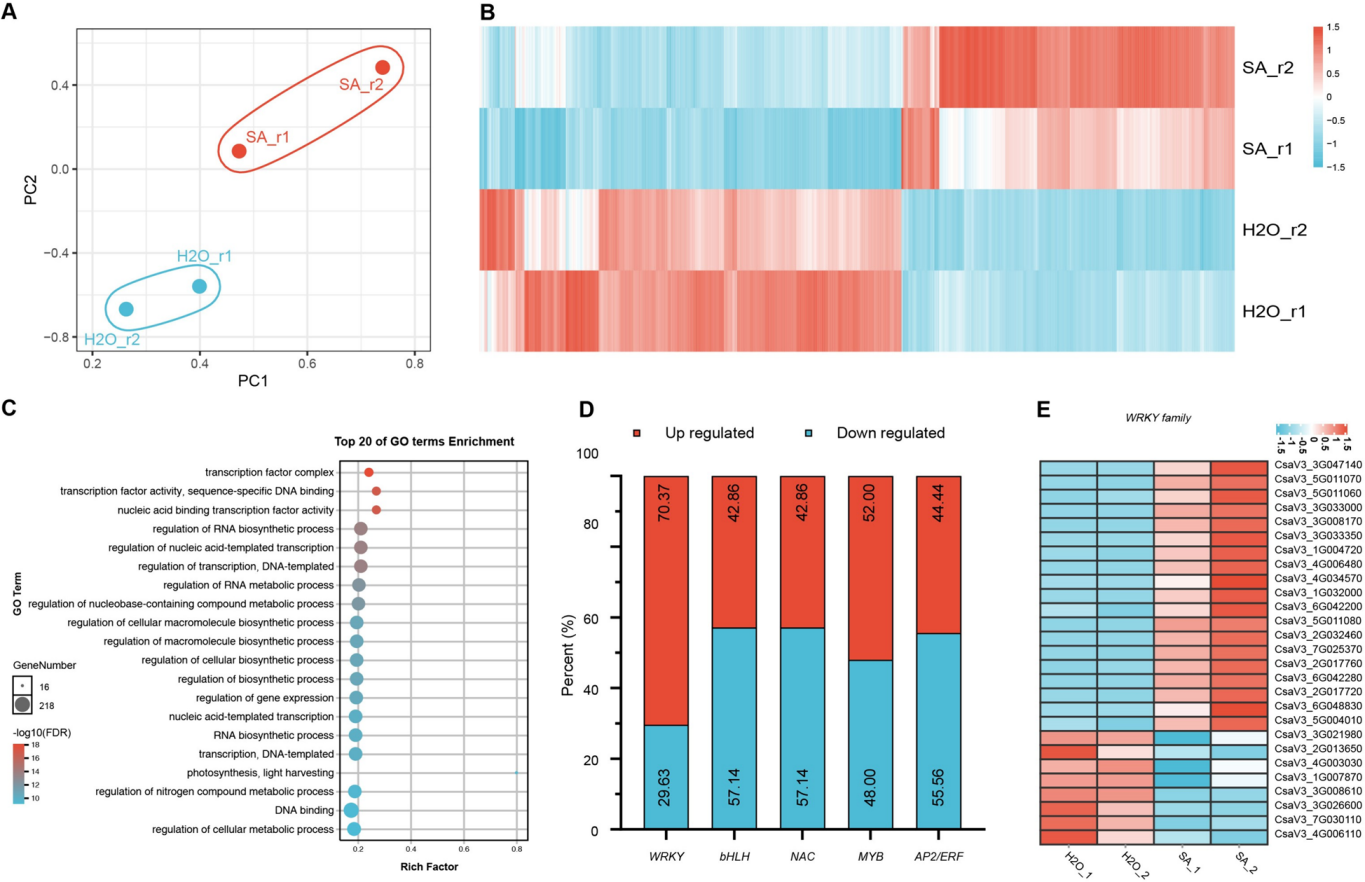
SA-activated signal transduction involves *WRKY* transcription factors. **A** Principal component analysis (PCA) of SA- and H_2_O-treated cucumber leaves. **B** The heatmap of DEGs between SA- and H_2_O-treated samples (*n* = 2 biological replicates). **C** GO analysis of SA-regulated DEGs. **D** A 100% stacked bar chart, showing the SA-regulated transcription factors in cucumber. **E** The heatmap of SA-regulated *WRKY* family genes in cucumber leaves. DEGs were defined on the basis of cut values Log_2_ (foldchange) ≥ 1, FDR < 0.05, samples were treated with SA and H_2_O for 30 min (*n* = 2 biological replicates)

In order to identify the early components of SA signaling pathway in cucumber, we analyzed a 30-min SA-induced transcriptome (Fig. 3A, B; Table S2). Gene Ontology (GO) analysis of SA-regulated differentially expressed genes (DEGs) revealed that these DEGs mainly enriched in the transcript regulation terms, e.g., transcription factor complex, transcription factor activity-sequence-specific DNA binding, nucleic acid binding transcription factor activity, regulation of RNA biosynthetic process, and regulation of nucleic acid-templated transcription, indicating that a short-period SA treatment strongly initiated the transcript regulation pattern in cucumber (Fig. 3C). *WRKY*, *bHLH*, *NAC*, *MYB*, *AP2/ERF* families are the biggest and most important plant transcription factor families. A further analysis of SA-induced DEGs showed that about 70% of *WRKY* transcription factors was up-regulated by SA treatment, in comparison to the situation in the families of *bHLH*, *NAC*, *MYB*, and *AP2/ERF*, where the percentages of SA-up-regulated vs down-regulated genes were more or less the same. The result likely suggests a substantial involvement of *WRKY* transcription factors in the pathway of SA signal transduction in cucumber (Fig. 3D, E).

### CsNPR1 robustly induces the expression of *CsWRKY11*

**Fig. 4:**
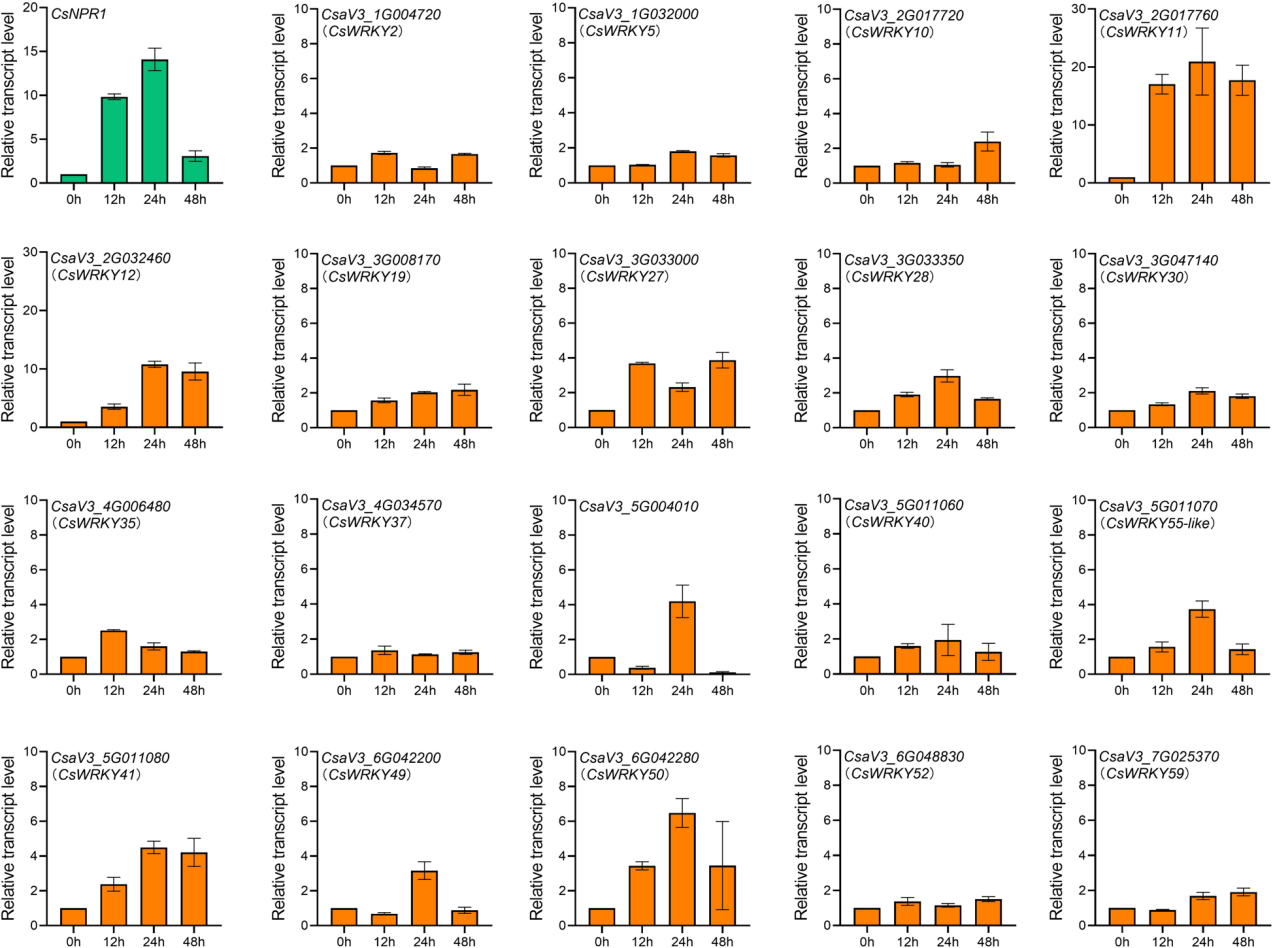
CsNPR1 regulates the expression of *CsWRKYs*. Time-course expression patterns of *CsWRKYs* after *CsNPR1* being over-expressed in cucumber cotyledons. Relative transcript levels were calculated as the ratio of those measured in *p35S::CsNPR1*- over those in empty vector-transformed cotyledons, *n* = 3 biological replicates

NPR1, an SA receptor as well as a co-activator, is the core component of SA signaling pathway, efficiently regulating downstream genes (Spoel et al. 2009; Ding et al. 2018). With the transiently over-expressed system which we build previously in cucumber cotyledons, we further examined whether SA-induced Cs*WRKYs* are the targets of CsNPR1 (Zhang et al. 2022a). As show in Fig. 4, the expression of *CsNPR1* increased gradually and reached a peak around 24-h time-point post infiltration (hpi). In the CsNPR1-overexpressed cotyledons, about half of the SA-induced Cs*WRKYs* were more or less up-regulated around 12 or 24 hpi, including *CsWRKY11* (*CsaV3_2G017760*), *CsWRKY12* (*CsaV3_2G032460*), *CsWRKY27* (*CsaV3_3G033000*), *CsWRKY28* (*CsaV3_3G033350*), *CsWRKY35* (*CsaV3_4G006480*), *CsWRKY41* (*CsaV3_5G011080*), *CsWRKY49* (*CsaV3_6G042200*), *CsWRKY50* (*CsaV3_6G042280*), *CsWRKY55-like* (*CsaV3_5G011070*) and *CsaV3_5G004010*, implying that these genes might be the targets of CsNPR1 (Fig. 4); the remaining half*,* in contrast, did not show obvious responses, including *CsWRKY2* (*CsaV3_1G004720*), *CsWRKY5* (*CsaV3_1G032000*), *CsWRKY10* (*CsaV3_2G017720*), *CsWRKY19* (*CsaV3_3G008170*), *CsWRKY30* (*CsaV3_3G047140*), *CsWRKY37* (*CsaV3_4G034570*), *CsWRKY40* (*CsaV3_5G011060*), *CsWRKY52* (*CsaV3_6G048830*) and *CsWRKY59* (*CsaV3_7G025370*), suggesting that their expression may not be mediated by CsNPR1. Notably, *CsWRKY11* (*CsaV3_2G017760*) was most dramatically up-regulated after *CsNPR1* being over-expressed, with its expression level reaching a peak around 24 hpi, which is dynamically consistent with the expression of *CsNPR1*. This finding likely suggests a crucial role of CsWRKY11 in CsNPR1-mediated SA signal transduction in cucumber.

### CsWRKY11 and CsNPR1 directly interact on the promoter of *CsWRKY11* to activate its transcription

**Fig. 5:**
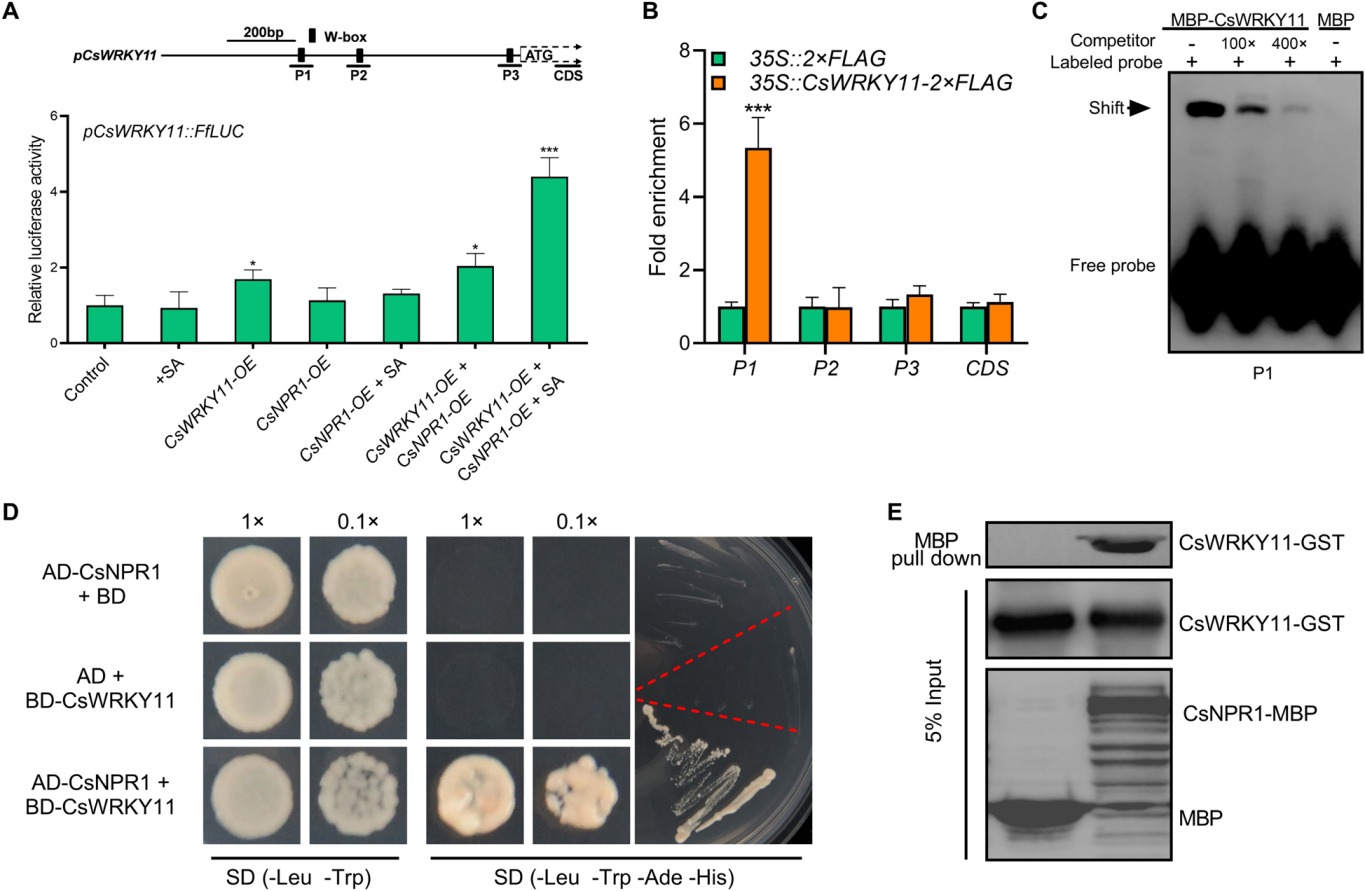
CsNPR1 promotes the transcriptional activation of CsWRKY11 on its promoter. **A** Dual-luciferase analysis of the effects of CsWRKY11 and CsNPR1 on the promoter activity of *CsWRKY11* (*pCsWRKY11*::*FfLUC*). Arabidopsis protoplasts were co-transformed with *pCsWRKY11*::*FfLUC* and *p35S*::*CsWRKY11*, *p35S*::*CsNPR1* or empty vector (control) alone or in combination, LUC activity was monitored 16 h post culturing. **B** ChIP-qPCR assay of the binding of CsWRKY11 to its encoding gene promoter. *p35S::CsWRKY11-2* × *FLAG-* and *p35S::2* × *FLAG*-transfected cucumber cotyledons were used for the analysis. Enrichment of the target fragments was normalized to *CsACTIN*, the coding region was used as an internal control. **C** EMSA of the interaction between CsWRKY11 and W-box on its encoding gene promoter. A *CsWRKY11* promoter fragment containing a W-box (P1) was biotin-labeled as a probe, with the same fragment unlabeled being used as a competitor. **D** Physical interaction of CsNPR1 and CsWRKY11 in the Y2H assay. The p*GADT7-CsNPR1* and p*GBKT7-CsWRKY11* plasmids were co- transferred into yeast strain AH109, and positive clones was grown and screened on a quadruple dropout (QDO: –Leu, –Trp, –Ade, –His) medium. p*GADT7-CsNPR1* + p*GBKT7* and p*GADT7* + p*GBKT7-CsWRKY11* transformed yeast cells were used as negative controls. **E** Physical interaction of MBP-CsNPR1 and GST-CsWRKY11 in the pull-down assay. Coding sequences of *CsNPR1* and *CsWRKY11* were inserted into pMAL-c5g and pGEX4T-1 plasmids respectively; plasmid-transformed *Escherichia coli* (BL21) cells were used for protein preparation. Input and pulled-down proteins were detected with anti-GST and anti-MBP antibodies

As an enhanced expression of CsNPR1 led to a robust up-regulation of *CsWRKY11*, we then set to determine whether CsNPR1 directly regulates the promoter activity of *CsWRKY11*. A dual-luciferase assay showed that over-expressed CsNPR1 alone was not able to activate the promoter activity of *CsWRKY11*. In contrast, over-expressed CsWRKY11 could significantly impose an activating effect on *CsWRKY11* promoter. Interestingly, the activating effect was greatly enhanced when CsNPR1 was co-expressed, particularly in the presence of SA (Fig. 5A). A motif screening was then carried out and three candidate W-boxes identified in the promoter region of *CsWRKY11* (Fig. 5A). Chromatin immunoprecipitation-qPCR (ChIP-qPCR) analysis revealed that CsWRKY11 preferentially bound to the first W-box (P1) in vivo (Fig. 5B). The direct interaction between CsWRKY11 and W-box (P1) was further validated by electrophoretic mobility shift assay (EMSA) in vitro (Fig. 5C). Considering that CsNPR1 and CsWRKY11 could synergistically enhance the activity of *CsWRKY11* promoter (Fig. 5A) and CsNPR1 could act as a co-activator, we further examined whether CsNPR1 directly interacts with CsWRKY11. In Y2H assay, only the yeast cells co-transformed with p*GADT7-CsNPR1* and p*GBKT7-CsWRKY11* could grow on quadruple dropout medium, indicating that CsNPR1 is able to directly interact with CsWRKY11 (Fig. 5D). This direct interaction between CsNPR1 and CsWRKY11 was further validated by a MBP (maltose binding protein) pull-down assay in vitro (Fig. 5E). Collectively, our analyses convincingly demonstrate that CsWRKY11 and CsNPR1 are able to directly interact on *CsWRKY11* promoter to synergistically activate its expression.

### CsNPR1 and CsWRKY11 synergistically promote SA-induced chlorophyll degradation and ROS burst

**Fig. 6:**
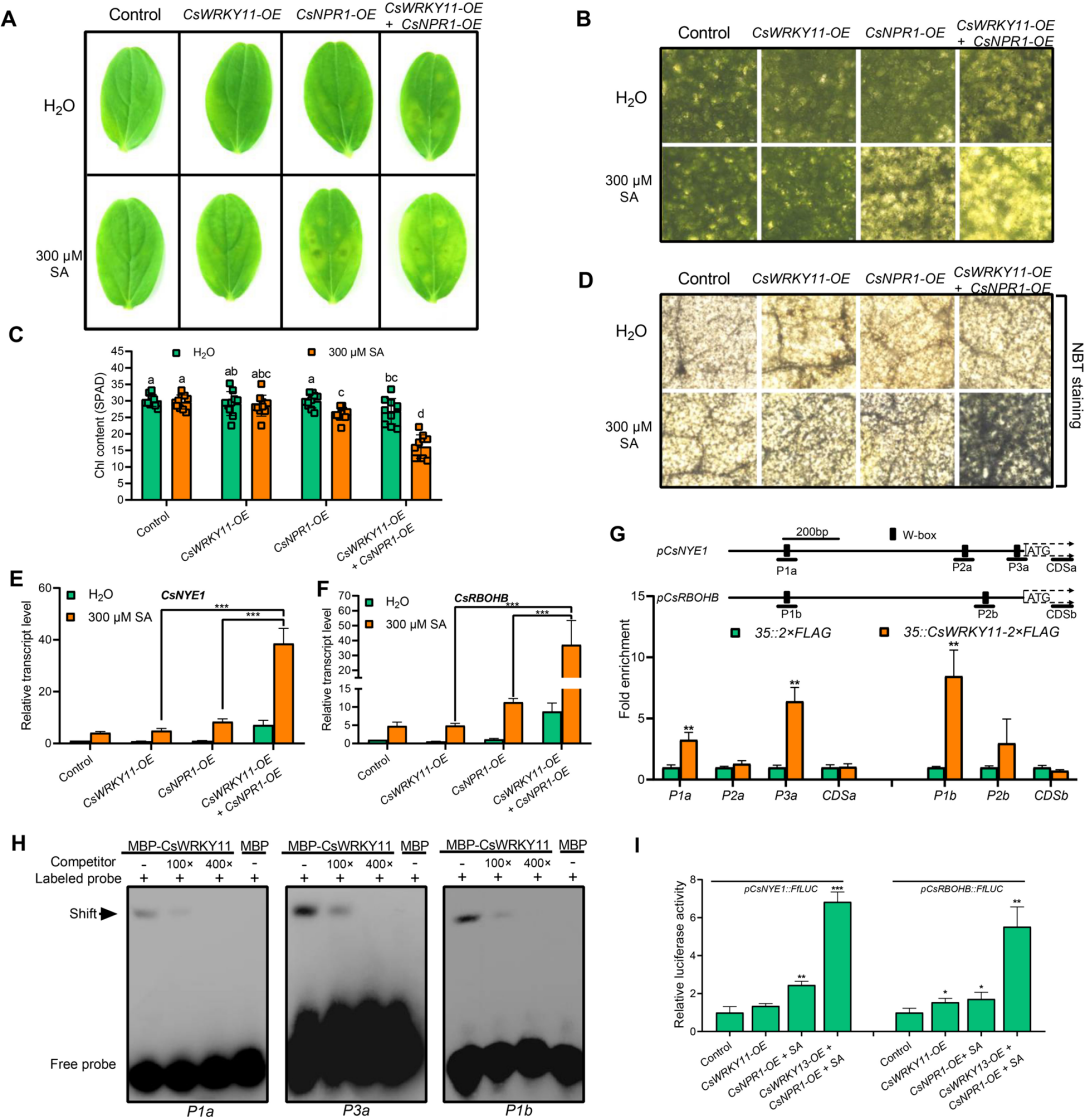
CsWRKY11 and CsNPR1 synergistically promote chlorophyll degradation and ROS biosynthesis. **A** De-greening phenotypes of cucumber cotyledons after *CsWRKY11* and/or *CsNPR1* being transiently over-expressed, in the presence or absence of SA. **B** Microscopic images of the leaf tissues around *Agrobacterium tumefaciens*-infiltrated sites on *CsWRKY11*- and/or *CsNPR1*-overexpressed cotyledons, in the presence or absence of SA. **C** Chlorophyll contents in *CsWRKY11*- and/or *CsNPR1*-overexpressed cucumber cotyledons, in the presence or absence of SA. **D** Microscopic images of the leaf tissues around *Agrobacterium tumefaciens*-infiltrated sites on *CsWRKY11*- and/or *CsNPR1*-overexpressed cotyledons following NBT staining, in the presence or absence of SA. **E** Relative transcript levels of *CsNYE1* in *CsWRKY11*- and/or *CsNPR1*-overexpressed cotyledons, in the presence or absence of SA. **F** Relative transcript levels of *CsRBOHB* in *CsWRKY11*- and/or *CsNPR1*-overexpressed samples, in the presence or absence of SA. In (**A-F**), 300 μM SA was used for treatment, cotyledons infiltrated with empty vector (*pCHF3*) transfected-*Agrobacterium tumefaciens* were used as controls. Cotyledon samples were photographed and then harvested for analysis four days post treatment. **G** ChIP-qPCR assay of the binding of CsWRKY11 to *CsNYE1* and *CsRBOHB* promoters. *p35S::CsWRKY11-2* × *FLAG-* and *p35S::2* × *FLAG*-transfected cucumber cotyledons were used for analysis. Enrichment of the target fragments was normalized to *CsACTIN*, the coding regions were used as internal controls. **H** EMSA of the interaction between CsWRKY11 and W-boxes on *CsNYE1* and *CsRBOHB* promoters. Promoter fragments containing W-boxes were biotin-labeled as probes, with the same fragments unlabeled being used as competitors. **I** Dual-luciferase analysis of the effects of CsWRKY11 and CsNPR1 on the activity of *pCsNYE1*::*FfLUC* and *pCsRBOHB*::*FfLUC*. *pCsNYE1*::*FfLUC* or *pCsRBOHB*::*FfLUC* was co-introduced into tobacco leaves with *p35S::CsWRKY11* or *p35S::CsNPR1* via the mediation of *Agrobacterium tumefaciens*, leaves transfected with *Agrobacterium tumefaciens* containing empty vectors were used as controls, SA treatment was implemented by spraying 300 μM SA solution onto the surface of tobacco leaves. Data are means ± SD (*n* = 3 biological replicates). *P < 0.05, **P < 0.01, ***P < 0.001 (*t*-test), and different letters indicate significant difference at *P* < 0.05 (one-way ANOVA test)

Inspired by the previous findings that SA induces both leaf de-greening and ROS burst, and more substantially, CsNPR1-CsWRKY11 molecular module mediates the early SA signal transduction (Figs. 1 and 5), we postulated that this molecular module might directly participate in regulating chlorophyll degradation and ROS biosynthesis. To verify the postulation, we over-expressed *CsNPR1* and/or *CsWRKY11* in cucumber cotyledons in presence or absence of SA or H_2_O. As shown in Fig. 6A-D, over-expressing *CsNPR1* or *CsWRKY11* alone was insufficient to induce the obvious alterations of physiological parameters, whereas over-expression of both *CsNPR1* and *CsWRKY11* together could moderately promote cotyledon de-greening and ROS accumulation. However, upon SA treatment, over-expression of *CsNPR1* could significantly induce cotyledon de-greening and ROS accumulation. As observed before, the induction was further enhanced when *CsWRKY11* was over-expressed simultaneously, although up-regulating *CsWRKY11* alone was insufficient to induce a similar phenotype. These results imply that *CsWRKY11* regulates chlorophyll degradation and ROS biosynthesis in a CsNPR1 dependent manner. Examination of the expression of marker genes involved in chlorophyll degradation and ROS biosynthesis pathways showed that, *CsNYE1* and *CsRBOHB* were both robustly up-regulated by a synergistic effect between CsWRKY11 and CsNPR1 (Fig. 6E and F). ChIP-qPCR analysis revealed that CsWRKY11 significantly bind to W-box-contained fragments, especially *P3a* located in *CsNYE1* promoter, and *P1b* located in *CsRBOHB* promoter in vivo (Fig. 6G). The direct interactions between these W-boxes and CsWRKY11 were further validated by EMSA (Fig. 6H). We then examined the effect of CsWRKY11 and CsNPR1 on the promoter activity of *CsNYE1* and *CsRBOHB*. Results showed that the effect of CsWRKY11 alone on the promoter activity of *CsNYE1* and *CsRBOHB* is limited. Interestingly, similar to the previous situation, CsWRKY11 strongly induced the promoter activity of both *CsNYE1* and *CsRBOHB* when CsNPR1 was co-introduced (Fig. 6I). These results demonstrate that CsWRKY11 and CsNPR1 are interdependent in regulating SA-mediated chlorophyll degradation and ROS biosynthesis processes.

## Discussion

**Fig. 7:**
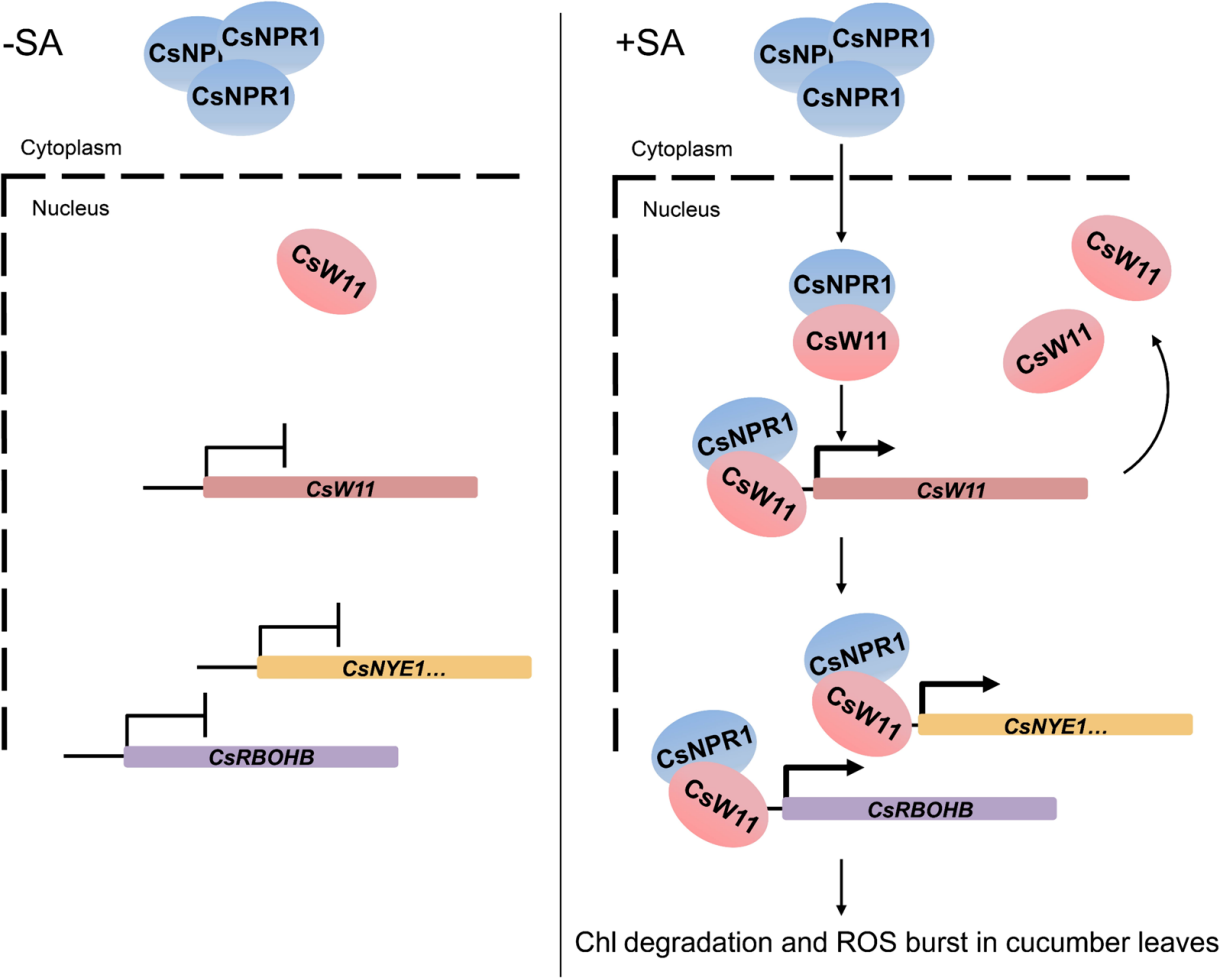
A proposed model of CsNPR1 and CsWRKY11 involved in synergistically regulating SA-triggered chlorophyll degradation and ROS production. In present of SA, CsNPR1 is depolymerized to monomer to enter the nucleus. Nucleus-located CsNPR1 rapidly recruits the transcription factor CsWRKY11 to bind to the W-box on the promoter of *CsWRKY11*; after amplifying the primary SA signal via self-activation, CsWRKY11 further effects synergistically with CsNPR1 to up-regulate the expression of chlorophyll degradation and ROS biosynthesis related genes, thereby leading to SA-triggered leaf de-greening and ROS burst in cucumber

SA is an important phytohormone, regulating not only plant immune response but also growth and development (Peng et al. 2021). Previous studies have made an enormous progress in the elucidation of SA signal transduction pathway, particularly in *Arabidopsis*; nevertheless, it still remains elusive as for how SA signal regulates the expression of downstream genes in cucumber (Lai et al. 2018; Li et al. 2018, 2022; Olate et al. 2018; Chen et al. 2019; Huang et al. 2020; Nomoto et al. 2021). NPR1 is the crucial component of SA signaling pathway, initially identified as a transcriptional co-activator, which itself lacks of DNA binding motif. NPR1 generally interacts with other transcription factors, like TGAs, to regulate SA responsive genes (Zhang et al. 1999; Peng et al. 2021; Kumar et al. 2022). In the present study, taking advantage of the research system which we built and verified in cucumber cotyledons before (Zhang et al. 2022a), we identified a WRKY transcription factor in cucumber, CsWRKY11, which physically associates with CsNPR1 to initiate SA signaling by activating the expression of its encoding gene (Fig. 5). Importantly, we further demonstrate that the CsNPR1-CsWRKY11 regulatory module synergistically up-regulates the chlorophyll degradation and ROS biosynthesis related genes (Fig. 6). ChIP-qPCR and EMSA analyses showed that CsWRKY11 directly bound to its target site (W-box), but overexpressing CsWRKY11 alone did not lead to a robust activation of its target promoter as well as a significant physiological alteration in cucumber (Figs. 5 and 6). Our analyses indicate that the trans-activating capability of CsWRKY11 is limited in the absence of CsNPR1, implying that CsWRKY11 might mainly act as an “anchor” to recognize and bind to its target sites. Co-overexpression of CsNPR1 and CsWRKY11 moderately up-regulated the expression of target genes, which was dramatically enhanced by SA treatment (Figs. 5 and 6). This observation further highlights that SA treatment-promoted depolymerization and nucleus localization of NPR1 are crucial for the activation of *CsWRKY11* on its target promoters, and only by forming a transcriptional regulation complex with CsNPR1 could CsWRKY11 efficiently regulate the expression of its target genes (Fig. 7).

WRKY transcription factors consist of many homologous proteins, many of which work redundantly in regulating common processes and responses (Wang et al. 2023). In our experiments, in addition to *CsWRKY11*, a few other *WRKYs*, like *CsWRKY12*, *CsWRKY27*, and *CsWRKY28* etc., were also found to be induced by SA treatment and over-expression of CsNPR1, implying that these *WRKYs* may work synergistically downstream of CsNPR1 on regulating SA-triggered responses (e.g., leaf senescence) (Figs. S1, S2, S3 and S4). On the hand, some SA-up-regulated *CsWRKYs*, including *CsWRKY2*, *CsWRKY5*, *CsWRKY10 CsWRKY19*, *CsWRKY30*, *CsWRKY37*, *CsWRKY40*, *CsWRKY52* and *CsWRKY59*, were not up-regulated by the over-expression of *CsNPR1*, suggesting that even though as an important hub regulator of SA signal, CsNPR1 is not always involved in regulating all the SA-induced *WRKYs* (Fig. 4). Actually, in addition to NPR1, NPR3 and NPR4 were also identified as SA receptors, mediating SA signal transduction in a negative mode, i.e. inhibiting the expression of downstream genes when being bound by SA (Fu et al. 2012; Ding et al. 2018). Therefore, those CsNPR1-uninducible *CsWRKYs* are likely regulated in the CsNPR3/4 dependent manner. We further hypothesize that this double regulatory mechanism may ensure an appropriate/unexcessive activation of SA signaling, at least to some extent.

Previous studies have extensively as well as intensively revealed that an elevated endogenous SA level and subsequent activation of SA signaling are closely correlated with plant defense responses (Ding and Ding 2020; Yan et al. 2020; Peng et al. 2021). In this study, we found that SA treatment promoted leaf senescence and ROS accumulation in cucumber (Fig. 1). ROS are the key element of plant immune system, which rapidly respond to pathogens’ attack and accumulate in pathogen-invaded tissues, thereby triggering hypersensitivity reactions (HR) (Glazebrook 2005; Yu et al. 2017). Even though not yet being precisely elucidated, increasing evidence supports that the regulatory process of plant immune process closely interconnects with that of leaf senescence (Zhang et al. 2020). Initiating leaf senescence is a strategy for plants to deal with stresses, and many senescence-associated genes have been shown to play an important role in plant immunity (Wang et al. 2020; Zhang et al. 2020). Our study convincingly demonstrates that crucial senescence genes (e.g., *CsNYE1*) and plant immunity gene *CsRBOHB* can be simultaneously induced by SA via the mediation of CsNPR1-CsWRKY11 module, which provides a new molecular basis to fully elucidate the interconnected regulation between plant immunity and leaf senescence.

In conclusion, the present study identifies a SA-triggered molecular regulatory pattern in cucumber, which involves a novel regulatory module, CsNPR1-CsWRKY11, directly regulating both a chlorophyll degradation gene and a ROS biosynthesis gene (Fig. 7). Our findings substantially contribute to the understanding of the interconnected regulation of not only between plant senescence and immunity, but also likely among other SA-induced responses in cucumber.

## Materials and methods

### Plant materials and growth conditions

*Cucumis sativus* L. ecotype Chinese Long 9930 was used for conducting all the experiments. Cucumber plants were cultured under the long‐day (16 h light/8 h dark) condition at 22 °C with a light intensity of 120 μmol m^−2^ s^−1^. Cucumber plants were planted in the soil mixed with peat soil, vermiculite, and pearlite.

### Chemical treatments

For SA treatment of cucumber leaves, detached cucumber leaves and cotyledons were floated on SA solutions with indicated concentrations; for SA treatment of tobacco leaves, living plants were sprayed with SA on the surface of *Agrobacterium tumefaciens*-infiltrated areas. In dual-luciferase analysis, SA was added to W5 solution before overnight culturing of plasmids-transfected protoplasts.

### Measurement of chlorophyll contents

Chlorophyll contents of cucumber leaves were determined with a SPAD‐502 PLUS chlorophyll meter.

### Measurement of Fv/Fm ratios

The LI‐6400 system (LI‐COR) was used to measure the maximal photochemical efficiencies of PSII (Fv/Fm) of cucumber leaves according to the manufacturer’s instructions.

### Measurement of ion leakage

A digital conductivity meter (Waterproof ECTestr11 + Multi-Range Tester) was used to measure the conductivity of cucumber leaves. Briefly, a sample was firstly immersed in deionized water and gently shaken at 25 °C for 60 min, and solution conductivity was then measured and recorded as C1. The sample was subsequently boiled for 15 min, and after cooling back to room temperature, the solution conductivity was measured and recorded as C2. The membrane ion leakage was calculated as the ratio of C1/C2.

### Measurement of ROS

Nitroblue tetrazoliun (NBT) staining was used for analyzing the content of O_2_^·−^ in cucumber organs. NBT was firstly dissolved in the phosphate buffer (pH 7.5) to the final concentration of 0.5 mg/ml to make a staining solution. Detached cucumber leaves and cotyledons were immersed in the staining solution, then vacuumed in the darkness for an hour, and finally boiled in 95% ethanol solution for decoloring.

### RNA extraction and RT-qPCR

Total RNAs were extracted using RNAiso Plus (Takara), and their cDNAs synthesized with a PrimeScript™ RT Master Mix kit (Takara). The quantitative real-time PCR (qPCR) was conducted with TB Green® Premix Ex Taq™ II (Tli RNaseH Plus) kit (Takara) on the CFX Connect Real-Time PCR Detection System (Bio-Rad). Primers for RT-qPCR are listed in Table S1.

### RNA-seq and data analysis

Total RNA extraction and sequencing (Illumina Novaseq6000/HiSeqX platform) were conducted by Novogene Bioinformatics Institute and Genergy Biotech Co. (Shanghai). The accession number of raw sequencing data is PRJNA771246 (SRA) (Zhang et al. 2022a). Genome data of cucumber and corresponding annotation were downloaded from http://cucurbitgenomics.org/organism/20 (Li et al. 2019). An RNA-seq analysis was performed with DESeq2 R package. DEGs were defined based on cutoff values of log_2_ (Fold Change) ≥ 1 and *P*-value < 0.01. PCA, GO enrichment, and heatmaps plots were performed by using gmodels, clusterProfiler, and pheatmap R packages, respectively. A cis-acting element analysis was carried out in https://meme-suite.org/meme/tools/fimo (Grant et al. 2011).

### Transient expression assays

The coding sequences of indicated genes were cloned and inserted into *pCHF3* vector for gene over-expression. The constructed plasmids were transferred into the *Agrobacterium tumefaciens* GV3101 strain, and the plasmid-containing *Agrobacterium tumefaciens* were then cultivated in YEB medium. The infiltration medium [0.5% sucrose, 10 mM MgCl_2_, 10 mM MES (pH = 5.6), 40 μl/L Silwet L-77, and 200 μM acetosyringone] was used to re-suspend cells to OD_600_ = 0.3. The re-suspended cells of *Agrobacterium tumefaciens* were placed under light for activation. Cucumber cotyledons were used to be infiltrated with a blunt-end syringe. After being infiltrated, cucumber plants were cultivated in a continuous light environment for gene overexpression.

### Chromatin immunoprecipitation assay

EpiQuik™ Plant ChIP Kit (Epigentek) with an anti‐Flag antibody was used for ChIP assay. CsWRKY11-2 × Flag was over-expressed in cucumber cotyledons using the transient expression system, and the protein-DNA complex was enriched with anti-Flag beads. The sample was cross-linked with 1% formaldehyde solution under vacuum for 20 min, and neutralized with 125 mM glycine for 10 min. After reverse cross-linking, the DNA fragments were extracted and cleaned with a ChIP DNA Clean & Concentrator kit (Zymo Research). Primers are listed in Table S1.

### Electrophoretic mobility shift assays (EMSA)

The coding sequence of *CsWRKY11* was inserted into pMAL-c5g vector and the resultant vector introduced into *E. coli* Rosetta (DE3) for protein expression. LightShift EMSA Optimization and Control Kit (ThermoFisher Scientific) was used to carry out EMSA, and unlabeled probes were used as competitors. A 15 μL reaction mixture was prepared by adding 1.5 μL 10 × binding buffer, 0.75 μg Poly (dI·dC), 1 μL of 50 fmol biotin‐labeled probes, indicated competitors, and 2 μg proteins. After 30 min incubation at 25 °C, the reaction mixture was electrophoresed with the polyacrylamide gel, and the separated DNAs and DNA–protein complexes were then transferred onto the nylon membrane for UV cross‐linking with a CL-1000 Ultraviolet Crosslinker. The Chemiluminescent Nucleic Acid Detection Module kit (ThermoFisher Scientific) was used to detect the biotin signals.

### Dual-luciferase reporter assays

For the *Arabidopsis* protoplast system, rosette leaves of 3-week-old *Arabidopsis* were used to prepare protoplasts. To-be-tested promoters were cloned into *pGreenII 0800-LUC* plasmid, while *CsWRKY11* and *CsNPR1* were cloned into *pCHF3* plasmid. The reconstructed *pGreenII 0800-LUC* and *pCHF3* plasmids were co-transferred into protoplasts via poly (ethylene glycol)-mediated transfection, and the transfected protoplasts were cultured at 22 °C overnight. Firefly and Renilla luciferase activities were then detected with the help of a dual-luciferase assay kit (Promega), and monitored with the Synergy two multi-mode microplate reader (Bio-Tek) according to the manufacturer’s instructions. For the living tobacco system, the reconstructed *pGreenII 0800-LUC* and *pCHF3* plasmids were respectively transferred into *Agrobacterium* strain *GV3101-pSoup-p19*. After activation under light, the plasmid-transformed *Agrobacterium* cells were co-infiltrated into the 4-week-old tobacco leaves. After a 24-h cultivation under light, *Agrobacterium* cells-infiltrated tissues were frozen in liquid nitrogen, and dual-luciferase reporter assays were performed using the same method as described in the *Arabidopsis* protoplasts system.

### Yeast two-hybrid (Y2H) assay

The CDS of *CsNPR1* and *CsWRKY11* were cloned into pGAD-T7 and pGBK-T7, respectively. The reconstructed plasmids were co-introduced into AH109 yeast cells. The transformed clones were firstly plated onto SD/‐Leu/-Trp medium for cultivation, and then they were plated onto SD/-Leu/-Trp/-Ade/-His medium for further analysis. p*GADT7-CsNPR1* + p*GBKT7* and p*GADT7* + p*GBKT7-CsWRKY11* transformed yeast cells were used as the negative controls.

### Pull down assay

Pull-down assay was performed as described previously (Zhang et al. 2021). Briefly, the CDS of *CsWRKY11* and *CsNPR1* were respectively cloned into p*GEX-4T-1* and p*MAL-c5G* plasmids. The reconstructed plasmids were then introduced into *Escherichia coli* Rosetta (DE3) cells for protein expression. The interacting protein complex was pulled down with anti-MBP Magnetic Beads (NEB), and the pulled-down proteins and input were examined with anti-GST and anti-MBP antibodies.

### Supplementary Information

**Additional file 1: Figure S1.** Phylogenetic tree of CsWRKY transcription factors. **Figure S2.** Protein interaction prediction of CsNPR1 and CsWRKY11. The protein interaction network was predicted and plotted in https://cn.string-db.org/. **Figure S3.** W-boxes enriched on the promoters of *SAGs*. **Figure S4.** Dual-luciferase analysis of the effects of CsWRKY11 and CsNPR1 on the promoter activity of *CsWRKYs*. Arabidopsis protoplasts were co-transformed with *pCsWRKYs*::*FfLUC *and *p35S*::*CsWRKY11*, *p35S*::*CsNPR1 *or empty vector (control) alone or in combination, SA was added to W5 solution before overnight culturing of plasmids-transfected protoplasts, LUC activity was monitored 16 hours post culturing. *P < 0.05, ***P < 0.001 (t-test).

**Additional file 2: Table S1.** Primers used in this work.

**Additional file 3: Table S2.** Gene expression information from transcriptome profiling.

## Data Availability

We confirmed that data supporting the results are available in the present article and its Supplementary Information.

## Abbreviations

CCEs: Chlorophyll catabolic enzymes
CCGs: Chlorophyll catabolic genes
ChIP: Chromatin immunoprecipitation
EMSA: Electrophoretic mobility shift assay
LUC: Firefly luciferaseqPCR: quantitative real-time PCR
REN: Renilla luciferase
ROS: Reactive oxygen species
RPKM: Reads per kilobase per million
SA: Salicylic acid
Y2H: Yeast two-hybrid
