# Supplementary material for: CsWRKY11 cooperates with CsNPR1 to regulate SA-triggered leaf de-greening and reactive oxygen species burst in cucumber
Source: Mol Hortic. 2024 May 22;4:21. doi: 10.1186/s43897-024-00092-5 (PMC11110285; doi:10.1186/s43897-024-00092-5)
Supplement: Supplementary file 1 — Additional file 1: Figure S1. Phylogenetic tree of CsWRKY transcription factors. Figure S2. Protein interaction prediction of CsNPR1 and CsWRKY11. The protein interaction network was predicted and plotted in https://cn.string-db.org/. Figure S3. W-boxes enriched on the promoters of SAGs. Figure S4. Dual-luciferase analysis of the effects of CsWRKY11 and CsNPR1 on the promoter activity of CsWRKYs. Arabidopsis protoplasts were co-transformed with pCsWRKYs::FfLUC and p35S::CsWRKY11, p35S::CsNPR1 or empty vector (control) alone or in combination, SA was added to W5 solution before overnight culturing of plasmids-transfected protoplasts, LUC activity was monitored 16 hours post culturing. *P < 0.05, ***P < 0.001 (t-test). [file 43897_2024_92_MOESM1_ESM.doc]

**SUPPORTING INFORMATION**

**CsWRKY11 cooperates with CsNPR1 to regulate SA-triggered leaf de-greening and reactive oxygen species burst in cucumber**

Dingyu Zhang^1, 2, #^, Ziwei Zhu^2, #^, Bing Yang^1^, Xiaofeng Li^1^, Hongmei Zhang^1^, and Hongfang Zhu^1*^

^1^Shanghai Key Laboratory of Protected Horticultural Technology, Horticultural Research Institute, Shanghai Academy of Agricultural Sciences, Shanghai 201403, China.

^2^State Key Laboratory of Genetic Engineering and Fudan Center for Genetic Diversity and Designing Agriculture, School of Life Sciences, Fudan University, Shanghai 200438, China.

^#^These authors contributed equally.

*****Corresponding author: zhuhongfang@saas.sh.cn (Hongfang Zhu).


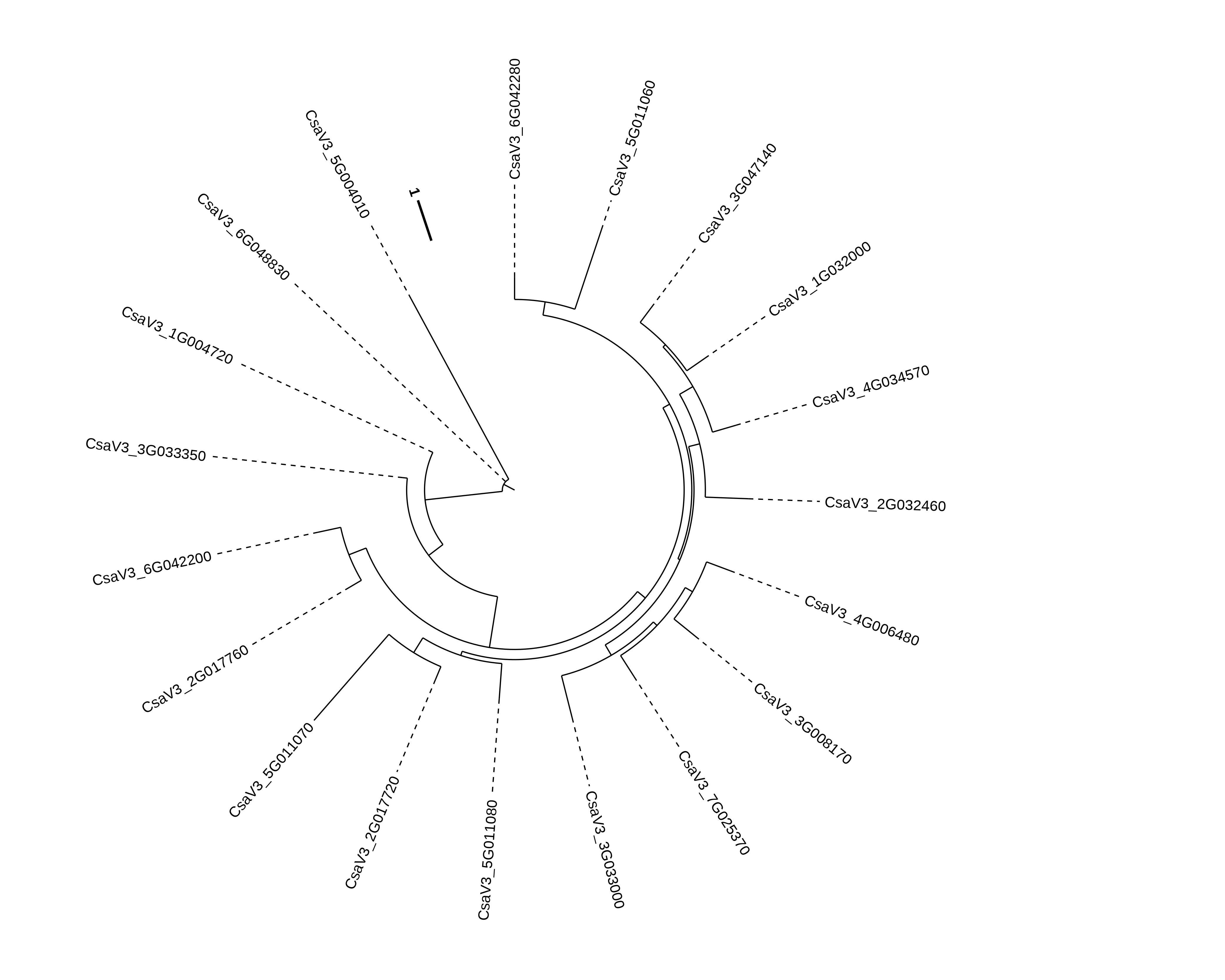


**Figure S1. Phylogenetic tree of CsWRKY transcription factors.**


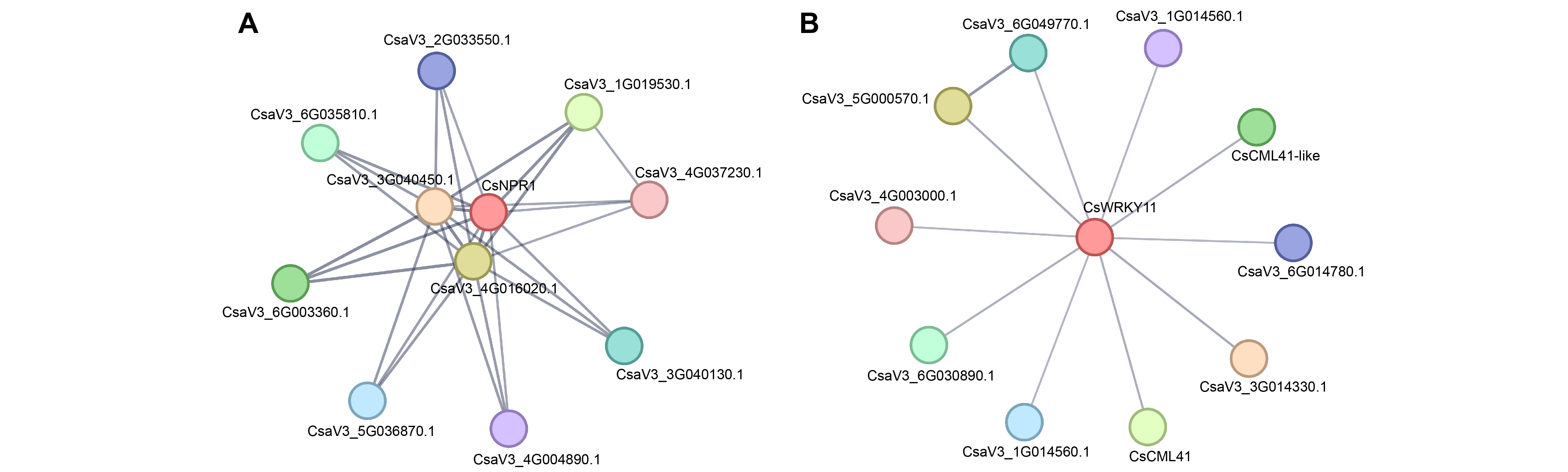


**Figure S2. Protein interaction prediction of CsNPR1 and CsWRKY11.**

The protein interaction network was predicted and plotted in https://cn.string-db.org/.


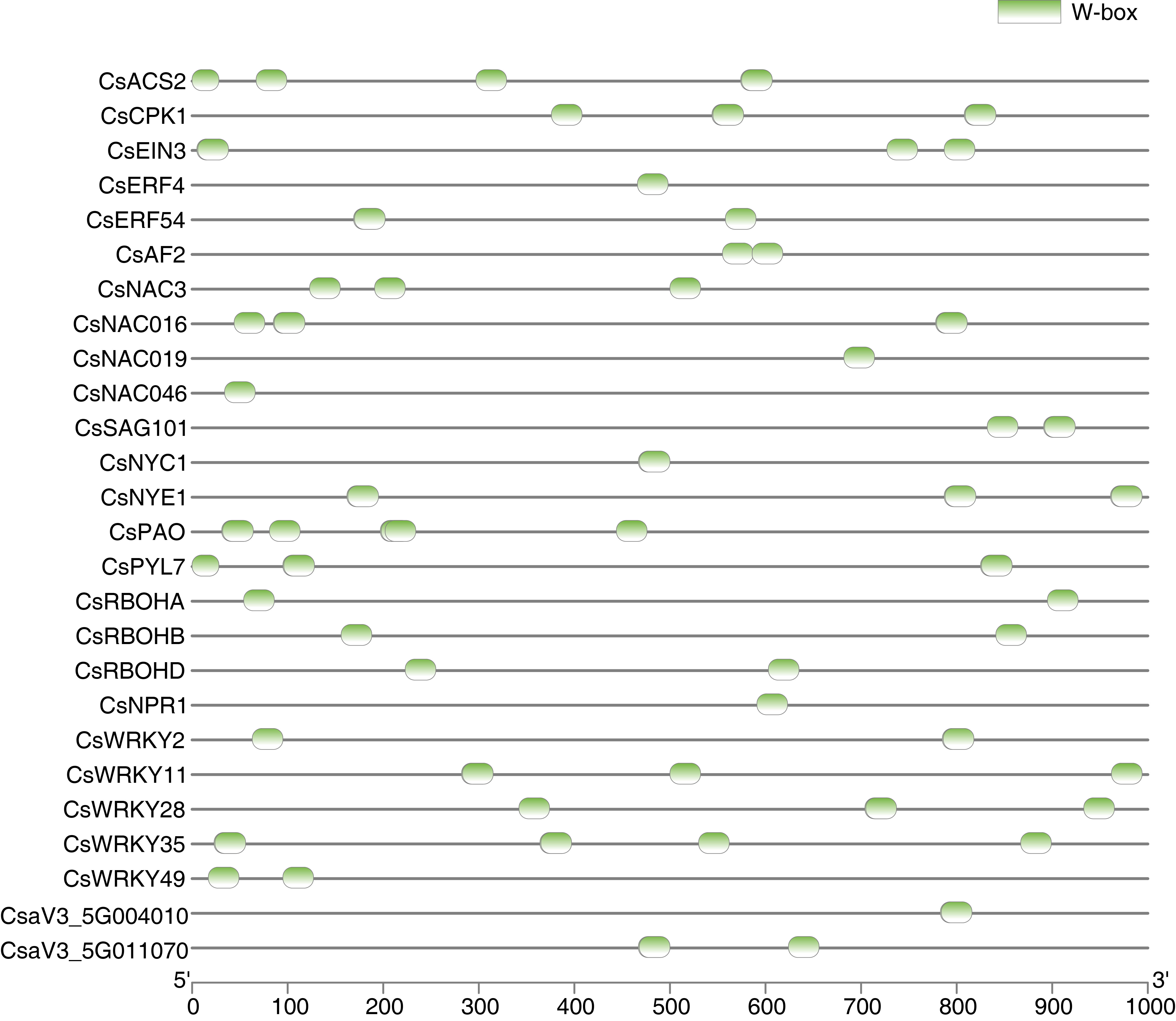


**Figure S3. W-boxes enriched on the promoters of *SAGs*.**


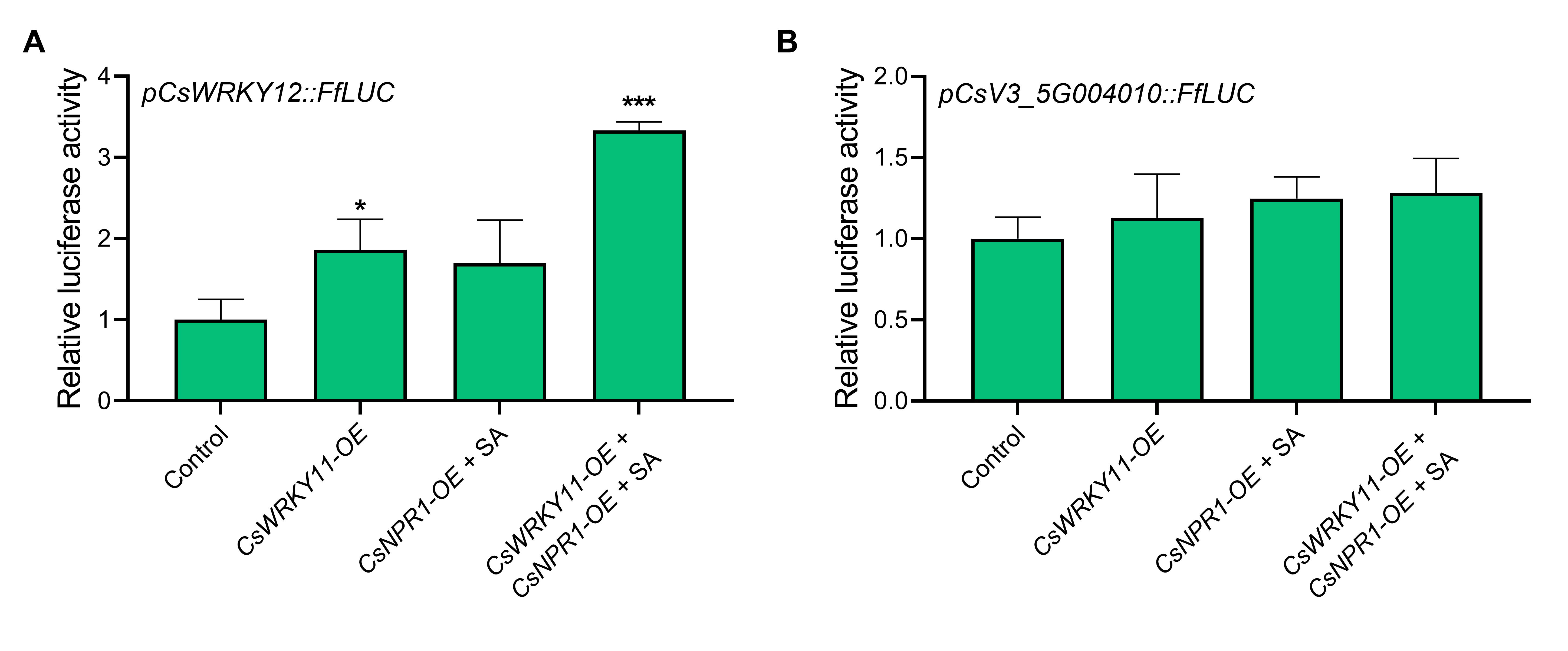


**Figure S4. Dual-luciferase analysis of the effects of CsWRKY11 and CsNPR1 on the promoter activity of *CsWRKYs*.**

Arabidopsis protoplasts were co-transformed with *pCsWRKYs*::*FfLUC* and *p35S*::*CsWRKY11*, *p35S*::*CsNPR1* or empty vector (control) alone or in combination, SA was added to W5 solution before overnight culturing of plasmids-transfected protoplasts, LUC activity was monitored 16 hours post culturing. **P* < 0.05, ****P* < 0.001 (t-test).

**Table S1. Primers used in this work.**

**Table S2. Gene expression information from transcriptome profiling.**
